# A Portable and Thermally Degradable Hydrogel Sensor Based on Eu-Doped Carbon Dots for Visual and Ultrasensitive Detection of Ferric Ion

**DOI:** 10.3390/molecules30153280

**Published:** 2025-08-05

**Authors:** Hongyuan Zhang, Qian Zhang, Juan Tang, Huanxin Yang, Xiaona Ji, Jieqiong Wang, Ce Han

**Affiliations:** 1 School of Science, Changchun Institute of Technology, 395 Kuanping Road, Changchun 130012, China; 2 School of Materials Science and Engineering, Changchun University, 6543 Weixing Road, Changchun 130022, China; 3 State Key Laboratory of Electroanalytical Chemistry, Changchun Institute of Applied Chemistry, Chinese Academy of Sciences, Changchun 130022, China

**Keywords:** carbon dots, fluorescent probes, hydrogel sensor

## Abstract

Degradable fluorescent sensors present a promising portable approach for heavy metal ion detection, aiming to prevent secondary environmental pollution. Additionally, the excessive intake of ferric ions (Fe^3+^), an essential trace element for human health, poses critical health risks that urgently require effective monitoring. In this study, we developed a thermally degradable fluorescent hydrogel sensor (Eu-CDs@DPPG) based on europium-doped carbon dots (Eu-CDs). The Eu-CDs, synthesized via a hydrothermal method, exhibited selective fluorescence quenching by Fe^3+^ through the inner filter effect (IFE). Embedding Eu-CDs into the hydrogel significantly enhanced their stability and dispersibility in aqueous environments, effectively resolving issues related to aggregation and matrix interference in traditional sensing methods. The developed sensor demonstrated a broad linear detection range (0–2.5 µM), an extremely low detection limit (1.25 nM), and rapid response (<40 s). Furthermore, a smartphone-assisted LAB color analysis allowed portable, visual quantification of Fe^3+^ with a practical LOD of 6.588 nM. Importantly, the hydrogel was thermally degradable at 80 °C, thus minimizing environmental impact. The sensor’s practical applicability was validated by accurately detecting Fe^3+^ in spinach and human urine samples, achieving recoveries of 98.7–108.0% with low relative standard deviations. This work provides an efficient, portable, and sustainable sensing platform that overcomes the limitations inherent in conventional analytical methods.

## 1. Introduction

Ferric ions (Fe^3+^) are widely recognized as one of the most common and biologically essential transition metals. In addition to their involvement in oxygen transport, enzyme catalysis, and hemoglobin synthesis, Fe^3+^ ions also participate in various other physiological processes [1,2]. It has been reported that Fe^3+^ can be taken into the human body through dietary intake or bioaccumulation in the food chain [3]. The accumulation of Fe^3+^ in excess can lead to a variety of health problems, including liver damage, oxidative stress, anemia, and neurodegenerative diseases such as Parkinson’s disease [4]. Further, the synthesis chlorophyll and protein in vegetables, along with their respiration rates under light and dark conditions, are closely correlated with Fe^3+^ concentrations in aquatic environments [5]. As a result, both from an environmental and a health perspective, it is important to develop rapid and efficient methods for detecting Fe^3+^ in order to minimize its harmful effects on ecosystems and human health. Although several analytical techniques such as atomic absorption spectroscopy (AAS) [6], inductively coupled plasma mass spectrometry (ICP-MS) [7], and electrochemical methods have been established [8], these traditional methods still face several scientific challenges. For example, they are generally unable to effectively detect trace amounts of Fe^3+^ in biologically relevant matrices with high ionic complexity; many detection platforms lack real-time response capabilities or require lengthy pretreatment [9]; current probes may have limited biocompatibility, environmental persistence, and be susceptible to photobleaching [10]. These issues highlight the need to design novel sensing platforms that are not only sensitive and highly selective, but also environmentally degradable, cost-effective, and easy to visualize and deploy in the field.

Due to their excellent optical properties, high environmental sensitivity, and ease of synthesis, carbon dots (CDs) have been extensively investigated for applications in sensing over the past few years [11]. CDs offer low toxicity, cost-effective preparation, and flexible surface modification, making them an attractive alternative to conventional quantum dots and organic dyes as zero-dimensional (0 D) carbon-based nanomaterials [12,13]. In addition to metal ions, pH values, small biomolecules, and bacteria, CD-based sensors have been successfully used to detect a variety of targets [14]. In neutral aqueous conditions, CDs tend to aggregate due to electrostatic attraction, hydrogen bonding, or covalent interactions, which results in fluorescence quenching [15,16]. Recent research has shifted attention to embedding CDs into biodegradable or thermoresponsive matrices, such as hydrogels. These hybrid platforms show great potential for portable, environmentally friendly, and low-pollution applications. However, despite significant progress, several challenges remain. For example, the reproducibility of carbon dot synthesis, particularly when using biomass-derived precursors, often leads to inconsistent optical output [17,18]. Furthermore, the interaction mechanisms between carbon dots and specific analytes (such as Fe^3+^), particularly in real sample matrices such as food or biological fluids, remain largely undefined. This is crucial for improving detection accuracy and avoiding false positives. As such, the development of a sensing platform based on host–guest interactions for the selective detection of specific heavy metal ions, while simultaneously preventing the self-aggregation of carbon dots, holds practical promise well.

Hydrogels possess a combination of structural and chemical characteristics such as cross-linking density, high porosity, and water affinity that render them highly compatible with carbon dot-based sensing platforms. They provide spatial confinement to embedded CDs, reducing diffusion and aggregation, which improves their stability and reproducibility [19,20]. A variety of hydrogel-based platforms, especially quasi-solid-state hydrogels, have been demonstrated to maintain the stability and dispersibility of CDs in aqueous environments. Compared with single-molecule fluorescent probes, CD@hydrogel systems offer enhanced structural integrity and environmental adaptability, enabling more stable and reusable sensing in complex media [21,22]. While single-molecule probes benefit from a small molecular size and good biocompatibility, they are often susceptible to photobleaching, irreversible degradation, and limited selectivity in harsh environments. In contrast, the hydrogel matrix not only physically protects embedded CDs but also permits the fine-tuning of sensor performance through chemical functionalization [23]. Recent studies have increasingly focused on the synergistic integration of CDs with biodegradable hydrogel matrices to create smart sensing platforms that combine responsiveness, environmental sustainability, and functional tunability. Such composites not only address the aggregation and fluorescence quenching issues commonly observed in aqueous environments but also exhibit enhanced mechanical flexibility and responsiveness to environmental stimuli such as pH, temperature, and ionic strength. Gong et al. developed a pH-responsive CDs@hydrogel that was employed in motion sensing and sweat monitoring [24]. The unique conductivity of CDs has also been utilized in portable fluorescent sensors for environmental monitoring and clinical diagnostics applications. Xie et al. fabricated a fluorescence sensor by incorporating CDs into an amphiphilic polyurethane matrix for Fe^3+^ detection, which demonstrated the potential for bioimaging and anti-counterfeiting applications [25]. Despite these advantages, there remain unresolved challenges, such as the precise control of fluorescence behavior within complex matrices, the reproducibility of large-scale fabrication, the quantitative correlation between visual colorimetric change and analyte concentration, and most existing hydrogel sensors are non-degradable [26], raising concerns regarding secondary environmental pollution after use [27]. Therefore, the development of degradable and environmentally friendly sensors is being actively pursued.

**Scheme 1:**
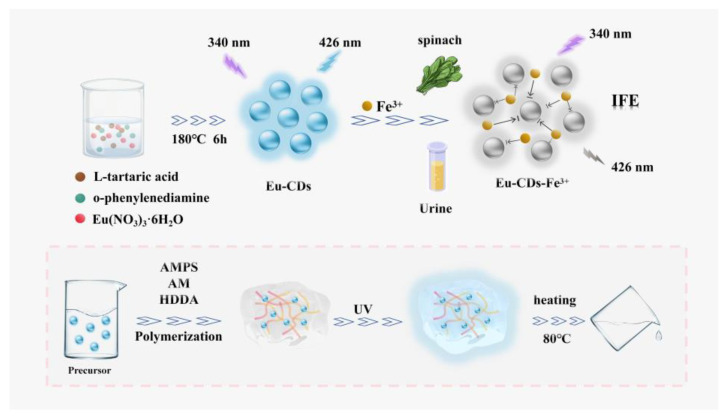
Eu-CDs as a fluorescent sensor for rapid and sensitive detection of Fe^3+^ in food and synthesis of CDs@DPPG.

In this study, a portable and thermally degradable fluorescent hydrogel sensor for the visual detection of Fe^3+^ has been developed, as illustrated in Scheme 1. Using L-tartaric acid and o-phenylenediamine as carbon and nitrogen sources, respectively, and europium nitrate hexahydrate as the Eu dopant, co-doped carbon dots (Eu-CDs) were synthesized. Eu-CDs emitted strong blue fluorescence, which was significantly quenched by Fe^3+^ via the inner filter effect (IFE). Eu-CDs were embedded through free radical polymerization into a degradable hydrogel matrix in order to build the sensing platform. 1,6-hexanediol diacrylate (HDDA) was employed as a crosslinker, potassium persulfate (KPS) as an initiator, and acrylamide (AM), and 2-acrylamido-2-methyl-1-propanesulfonic acid (AMPS) were used as monomers. The resulting hydrogel (Eu-CDs@DPPG) was shown to allow the smartphone-assisted visual detection of Fe^3+^ under UV light and could be thermally degraded after use, offering a convenient and environmentally friendly sensing solution.

## 2. Results

### 2.1. Synthesis and Characterization of Eu-CDs

Eu-CDs were synthesized via a one-step hydrothermal method using o-phenylenediamine and L-tartaric acid as carbon and nitrogen sources, respectively, while Eu(NO_3_)_3_·6H_2_O was employed as the dopant. The reaction conditions, including temperature and reaction time, were optimized (Figure S1). Under optimal conditions, the absolute quantum yield of the as-prepared Eu-CDs was determined to be 23.77%.

**Figure 1:**
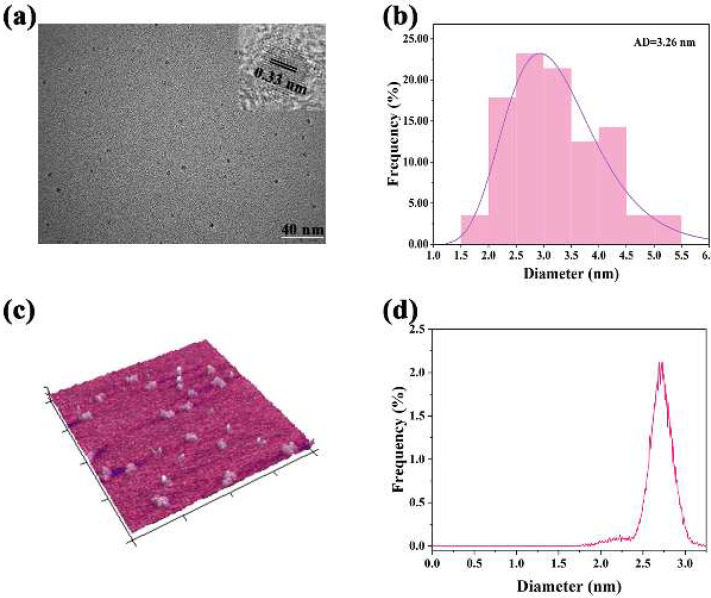
(**a**) TEM and HR-TEM images, (**b**) particle size distribution, (**c**) AFM image, and (**d**) height distribution of Eu-CDs.

Transmission electron microscopy (TEM) and high-resolution TEM (HR-TEM) were used to investigate the morphology and size of Eu-CDs. As shown in the TEM images (Figure 1a,b), the Eu-CDs exhibited good water dispersibility, a uniform spherical shape, and narrow size distribution. The average particle size, based on the measurement of 50 individual particles, was calculated to be 3.26 ± 0.36 nm. The HR-TEM image (inset of Figure 1a) revealed a distinct lattice spacing of 0.33 nm. In addition, atomic force microscopy (AFM) measurements showed that the height of Eu-CDs ranged from 1.77 to 3.25 nm, with an average thickness of 2.72 nm, corresponding to approximately 2–5 layers of stacked graphene sheets (Figure 1c,d), which was consistent with the TEM results.

**Figure 2:**
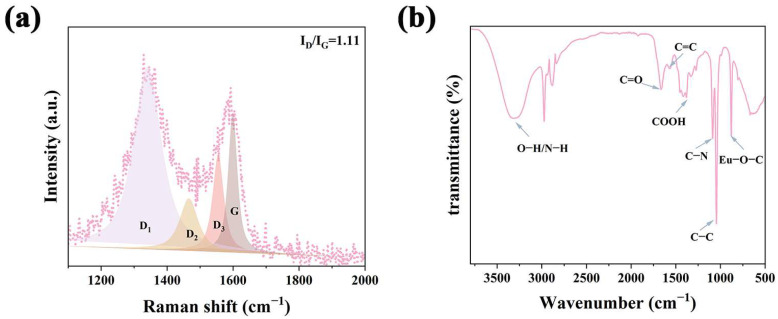
(**a**) Raman spectrum, (**b**) FT-IR spectrum of Eu-CDs.

Raman and Fourier transform infrared (FT-IR) spectroscopy were performed to further investigate the structural characteristics of Eu-CDs. In the Raman spectrum (Figure 2a), two prominent bands were observed at 1341 cm^−1^ and 1592 cm^−1^, corresponding to the D and G bands, respectively, which are associated with sp^3^ defects and sp^2^ carbon vibrations. The deconvolution of the Raman spectrum in the 1100–2000 cm^−1^ region revealed multiple sub-peaks. The peak at 1338 cm^−1^ (D_1_) was attributed to the edge bonding of sp^2^/sp^3^ hybridized carbon atoms [28], while the D_2_ band at 1466 cm^−1^ was assigned to -COOH or epoxy C-OH groups. The D_3_ band at 1553 cm^−1^ was associated with C=O and C-O moieties within the structure [29]. FT-IR analysis (Figure 2b) provided additional insight into the surface functional groups. It was determined that the broad peak at 3304 cm^−1^ was attributed to stretching vibrations N-H and O-H. The peaks at 1667 cm^−1^ and 1379 cm^−1^ were attributed to stretching vibrations C=O and -COOH [30,31]. The characteristic peaks at 1089 and 881 cm^−1^ were associated with C-N and Eu-O-C vibrations [32], respectively, which together contributed to the excellent water dispersibility of Eu-CDs.

**Figure 3:**
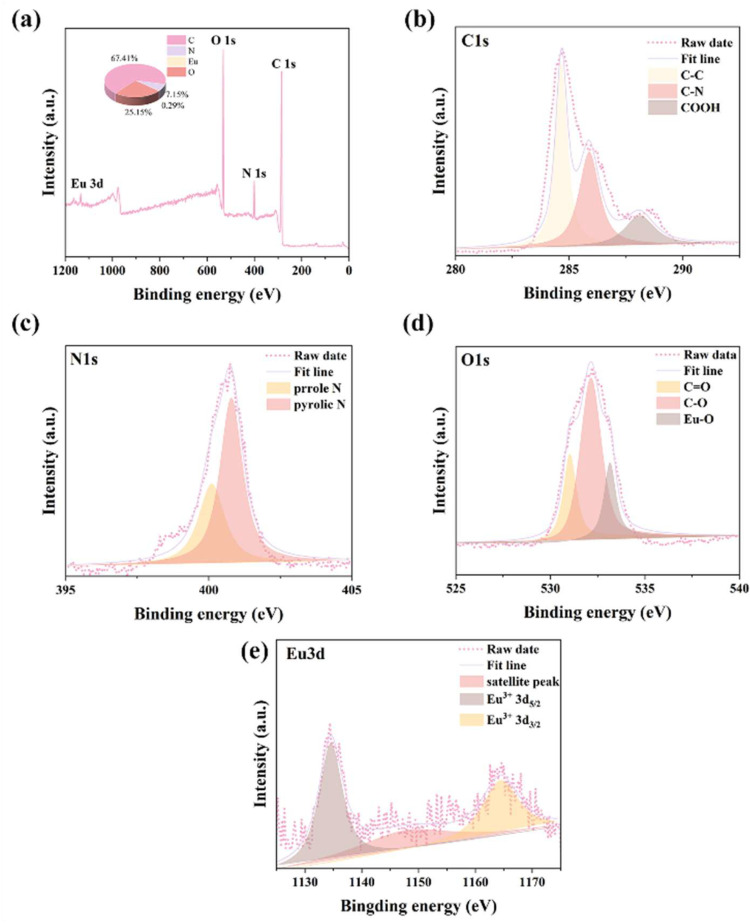
(**a**) XPS spectrum of Eu-CDs. The high-resolution XPS spectra of (**b**) C 1s, (**c**) N 1s, (**d**) O 1s, and (**e**) Eu 3d.

The chemical composition and surface functionalities were further confirmed by X-ray photoelectron spectroscopy (XPS). The survey spectrum (Figure 3a) showed the presence of C 1s (285.00 eV), N 1s (401.00 eV), O 1s (532.00 eV), and Eu 3d (1134.00 eV), with atomic percentages of 67.42%, 7.15%, 25.15%, and 0.29%, respectively. High-resolution C 1s spectra revealed peaks corresponding to C-C (284.65 eV), C-N (285.88 eV), and -COOH (288.07 eV) (Figure 3b) [33]. The N 1s spectrum displayed signals for both C-N (400.01 eV) and N-H (400.80 eV) bonds (Figure 3c). Deconvolution of the O 1s spectrum indicated the presence of C=O (531.05 eV), C-O (532.15 eV), and Eu-O (533.15 eV) groups (Figure 3d). Additionally, the Eu 3d spectrum confirmed the successful doping of Eu^3+^ into the CDs (Figure 3e) [34]. These XPS findings were in good agreement with the FT-IR results.

### 2.2. Optical Properties of Eu-CDs

The UV–Vis absorption spectrum of Eu-CDs showed two peaks at 242 and 270 nm, corresponding to n–π* and π–π* transitions, respectively (Figure S2a). Under excitation with a handheld UV lamp at 365 nm, Eu-CDs exhibited bright blue fluorescence (inset, Figure S2a). The maximum excitation and emission wavelengths were found to be 340 nm and 426 nm, respectively. As shown in Figure S2b, the emission peak red-shifted gradually with increasing excitation wavelength (300 to 400 nm), indicating an excitation-dependent behavior. This character was due to the presence of Eu atoms, which changed the size, surface state, and functional groups of the CDs. To evaluate their applicability, the fluorescence stability of Eu-CDs was tested under various conditions. The fluorescence remained stable even in the presence of 1.0 M NaCl (Figure S3a), suggesting excellent resistance to ionic interference. In addition, Eu-CDs exhibited strong thermal stability, maintaining fluorescence intensity after heating at 50 °C for 10 min (Figure S3b), as well as high photostability under continuous UV irradiation for 110 min (Figure S3c). The pH stability test showed a significant drop in fluorescence only under strongly alkaline conditions (pH = 12–14), which was attributed to the deprotonation of surface groups (Figure S3d) [35]. These results confirm the suitability of Eu-CDs for stable detection across a wide range of sample matrices.

### 2.3. Detection of Fe^3+^

**Figure 4:**
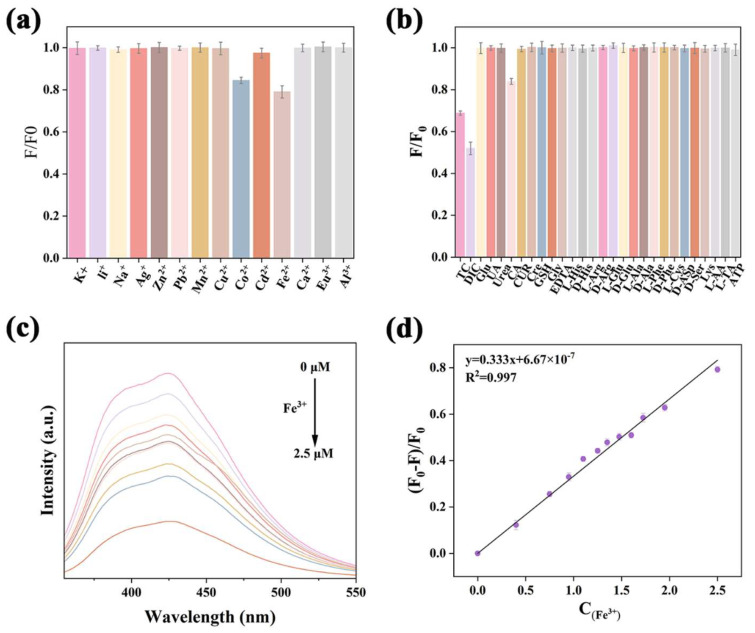
(**a**) The fluorescence intensity of Eu-CDs in the presence of different metal ions, (**b**) different common small molecules, the concentration was 0.1 M, (**c**) the fluorescence variation in Eu-CDs when exposed to Fe^3+^, (**d**) The relationship between fluorescence intensity and Fe^3+^.

Human health disorders, including thalassemia, hemochromatosis, and thyroid dysfunction caused by excessive Fe^3+^ intake in food, have been linked to abnormal Fe^3+^ concentrations in the body. Therefore, it was important to develop a reliable and selective detection method for Fe^3+^ [36]. For evaluating the selectivity of Eu-CDs, the fluorescence response of the sensor was assessed against various metal ions, and common biomolecules and inorganic substances in plants. According to Figure 4a,b, a significant decrease in fluorescence intensity was observed only in the presence of Fe^3+^, whereas other ions caused negligible changes, indicating that Fe^3+^ was well selected.

Additional metal ions were introduced into the Eu-CDs–Fe^3+^ system to examine possible interferences further. Despite the presence of these ions, the fluorescence remained relatively unaffected, indicating that the quenching effect was primarily caused by Fe^3+^ (Figure S4a). Also evaluated were the effects of pH on the fluorescence signal in the pH range of 1–11. Figure S4b showed that no significant fluctuations in fluorescence were observed under mildly acidic to weakly basic conditions, suggesting that pH-dependent deprotonation was not significantly affected by the detection system. A concentration-dependent quenching behavior was clearly observed when increasing amounts of Fe^3+^ (0–2.5 μM) were added to the Eu-CDs solution (Figure 4c). The fluorescence intensity decreased linearly with Fe^3+^ concentration, following the equation y = 0.333x + 6.67 × 10^−7^ with a correlation coefficient of R^2^ = 0.997 (Figure 4d). Based on the equation LOD = 3σ/k (where σ is the standard deviation of blank measurements and k is the slope of the calibration curve), the detection limit was calculated to be 1.25 nM. Moreover, the sensor responded rapidly, with fluorescence quenching observed within 40 s, demonstrating that the probe could be used for real time monitoring (Figure S5). In summary, these results demonstrate that Eu-CD probes were highly sensitive and selective toward Fe^3+^, making them suitable for practical sensing applications.

### 2.4. Proposed Sensing Mechanism

**Figure 5:**
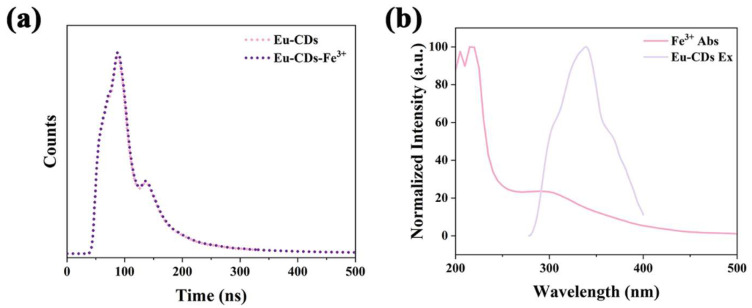
(**a**) Lifetimes of the Eu-CDs in the absence and presence of Fe^3+^, (**b**) the excitation spectra of the Eu-CDs and the UV–vis absorption of Fe^3+^.

To explore the fluorescence quenching mechanism of Eu-CDs by Fe^3+^, fluorescence lifetime measurements were first carried out. As shown in Figure 5a, the average lifetime of Eu-CDs before and after Fe^3+^ addition were nearly unchanged (τ_0_ = 3.71 ns, τ_1_ = 3.59 ns, τ_0_/τ_1_ = 1.03). The fluorescence lifetimes of Eu-CDs before and after the addition of Fe^3+^ were measured, and the corresponding average values, standard deviations (SD), relative standard deviations (RSD), t-values, and *p*-values (*p* > 0.05) were calculated to further support our conclusion (Table S1). It was indicated that neither dynamic quenching nor Forster resonance energy transfer (FRET) occur upon the addition of Fe^3+^ to the Eu-CDs solution. Further evidence was obtained from UV–Vis. Fe^3+^ did not produce any new absorption peaks when added to Eu-CDs, but there was significant overlap between the absorption spectrum and Eu-CDs excitation spectrum (Figure 5b). The calculation confirmed by Equation (S1) of the overlap integral J (J = 1.891 × 10^9^ nm^5^ × M^−1^ × cm^−1^) between the UV absorption spectrum of Fe^3+^ and the fluorescence excitation spectrum of Eu-CDs also confirmed that the inner filter effect (IFE) was the cause of fluorescence quenching (Figure S6). This was further confirmed by calculating the Parker Equations (S2)–(S4). The increasing values of F_obsd_/F_cor_ (3.466 to 4.196), E_obsd_ (0 to 0.242), and E_cor_ (0 to 0.374) indicated that IFE was the dominant quenching mechanism (Table S2). These results suggested that fluorescence quenching was mainly caused by IFE rather than static quenching.

### 2.5. Real Sample Analysis

To verify the practical applicability of the proposed sensing method, spinach and human urine were selected as real-world samples for Fe^3+^ detection in this study. Human urine has been reported to reflect systemic iron metabolism and can assist in the diagnosis of iron-related disorders, such as iron overload or renal dysfunction [37]. Meanwhile, spinach is a well-known dietary source of iron, and assessing its Fe^3+^ content helps to evaluate the iron enrichment level and nutritional value of foods, making it a relevant model for detecting iron content in food matrices [38]. Therefore, we spiked spinach and urine samples with Fe^3+^ at three concentrations (0.5, 1.5, and 2.5 μM). Each concentration was tested in triplicate. Fluorescence intensities were recorded, and recovery rates calculated (Table 1). The results showed acceptable recovery values ranging from 98.70% to 108.00%, with RSD as low as 1.05% to 2.17% which indicated that the method works reliably even in complex matrices. The proposed method was compared with others for detecting Fe^3+^ in terms of sample complexity and LOD. The results demonstrated that this method was not only suitable for more complex samples but also exhibited a low LOD and a wide linear range (Table S3). The applicability, anti-interference ability, and detection accuracy of Y-CDs in complex biological and food matrices were verified, providing a potential strategy for its practical application in food safety and clinical monitoring.

### 2.6. Portable and Self-Degradable Eu-CDs@DPPG Sensor

**Figure 6:**
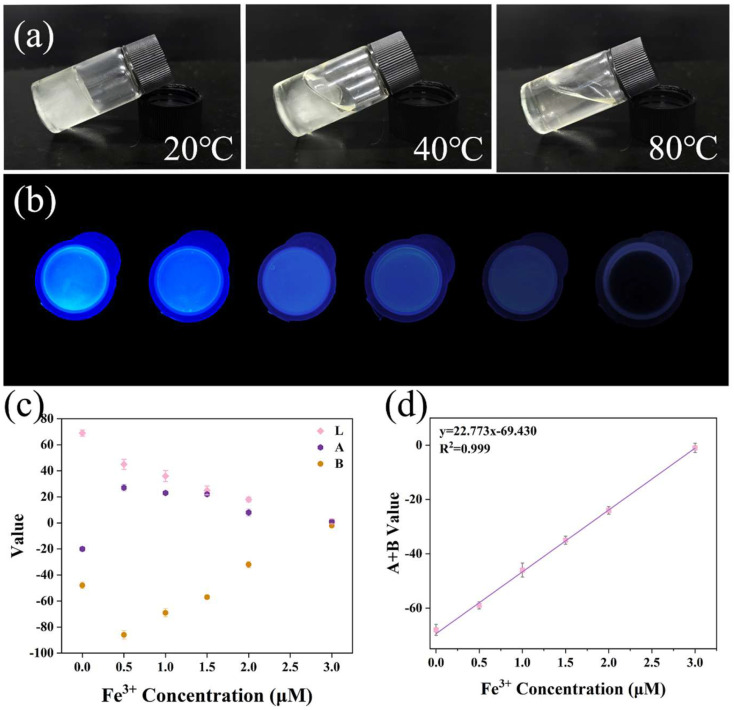
(**a**) Bottle test of the spontaneous degradation process of Eu-CDs@DPPGs from 20 to 80 °C, (**b**) photographs of Eu-CDs@DPPGs immersed in different concentrations of Fe^3+^, (**c**) relationship between LAB value and Fe^3+^ concentration, (**d**) linear relationship between A + B value and Fe^3+^ concentration.

Despite the widespread study of hydrogel-based Fe^3+^ sensors, the environmental safety of most sensors is limited by their degradability. In this work, Eu-CDs@DPPG composite is thermally degradable. As shown in Figure 6a, the bottle-test results demonstrated that when the hydrogel matrix (DPPG) was heated to 80 °C, the Eu-CDs@DPPG transitioned from a solid gel into a low-viscosity, flowable liquid, directly confirming its degradability. Furthermore, the monomers used (AM and AMPS) were highly hydrophilic, facilitating Fe^3+^ diffusion into the sensor and Eu-CD interaction within the polymer network. To evaluate the influence of hydrogel incorporation on the fluorescence behavior of Eu-CDs, we examined the variations in their LAB and RGB values after embedding them into the DPPG (Figure S7 and Table S4). The results revealed only slight changes in both fluorescence intensity (L channel) and emission color (RGB values), demonstrating that the fluorescence emission characteristics of Eu-CDs were largely preserved following incorporation into DPPG. In the presence of 365 nm UV light, Eu-CDs@DPPG exhibit fluorescence quenching as Fe^3+^ concentration from 0 increased to 3 μM (Figure 6b). ColorSnap was used to analyze the image quantitatively, and the extracted LAB values showed a linear relationship between the A + B channel ratio and Fe^3+^ concentration (y = 22.773x − 69.430, R^2^ = 999) (Figure 6c,d). Based on the equation LOD = 3σ/k (where σ is the standard deviation of blank measurements and k is the slope of the calibration curve), the detection limit was calculated to be 6.588 nM. These results confirm that Eu-CDs@DPPG hydrogel retained excellent sensing performance, while being portable and thermally degradable, demonstrating its potential for on-site Fe^3+^ monitoring.

## 3. Materials and Methods

The Materials and Reagents section, instrumental details, and assays for Fe^3+^ detection are provided in the Supplementary Materials.

### 3.1. Chemicals and Materials

o-Phenylenediamine, L-tartaric acid, europium(III) nitrate hexahydrate (Eu(NO_3_)_3_·6H_2_O), and all interfering ions and biomolecules used in this study were purchased from Aladdin (Shanghai, China). Ultrapure water used throughout the experiments was prepared with a Milli-Q Gradient system (Millipore, Burlington, MA, USA).

### 3.2. Instrumentation

The morphology and crystalline structure of the samples were examined using high-resolution transmission electron microscopy (JEOL-2100F, JEOL, Tokyo, Japan). UV–Vis absorption spectra were recorded with a spectrophotometer (Varian Cary 50, Agilent Santa, USA). FT-IR spectra were obtained using a spectrometer (IS50, Thermo, Waltham, MA, USA). Raman spectra were measured with a LABRAM HR Evolution system (LabRAM HR Evolutio, HORIBA, Irvine, CA, USA). X-ray diffraction (XRD) patterns were acquired using Cu Kα radiation on a diffractometer (RIGAKU D/MAX 2500, Rigaku, Tokyo, Japan). X-ray photoelectron spectroscopy (XPS) measurements were performed using a monochromated Al Kα source on a diffractometer (ESCALAB Mk II, VG Scientific, Waltham, MO, USA). Fluorescence excitation and emission spectra were collected using a spectrometer (LS-55, PerkinElmer, Bridgeport Avenue, CT, USA). Absolute photoluminescence quantum yields (Φ) were measured using a spectrofluorometer (QuantaMaster 8000, HORIBA, Irvin, CA, USA).

### 3.3. Synthesis of Eu-CDs

Eu-CDs were synthesized via a one-pot hydrothermal method. Briefly, L-tartaric acid (40 mM), o-phenylenediamine (20 mM), and Eu(NO_3_)_3_·6H_2_O (0.67 mM) were dissolved in 20 mL of ultrapure water. The mixture was transferred into a 50 mL Teflon-lined stainless-steel autoclave and heated at 200 °C for 8 h. After cooling to room temperature naturally, the solution was filtered through a 0.22 μm membrane to remove insoluble carbonized materials. The filtrate was dialyzed (MWCO: 1000 Da) against ultrapure water for 6 h, yielding a yellow Eu-CDs solution. The product was lyophilized to obtain dry Eu-CDs powder, which was redispersed in water for subsequent use.

### 3.4. Stability Evaluation of Eu-CDs

To evaluate the environmental stability of Eu-CDs, 1 mg/mL solution was used in all tests. Fluorescence spectra were measured after adding various concentrations of NaCl and H_2_O to determine ionic strength tolerance and oxidative stability. By adjusting Eu-CDs solution pH (0.2 mg/mL) from 1.0 to 14.0 and recording the corresponding fluorescence intensity, pH stability was evaluated. Incubation of 3 mL of Eu-CDs solution at temperatures ranging from 30 to 50 °C for 10 min was used for thermal stability evaluation. For photostability evaluation, 365 nm UV light was used to illuminate the solution and monitor changes in fluorescence over a period of 50 min. Fluorescence spectra were also observed under excitation wavelengths ranging from 300 to 400 nm in order to evaluate the excitation-dependent emission behavior.

### 3.5. Detection of Fe^3+^

For fluorescence detection in the solution, 2.9 mL of Fe^3+^ solution with different concentrations (0–2.5 μM) was mixed with 100 μL of diluted Eu-CDs solution (1 mg/mL). After reacting for 60 s at room temperature, the fluorescence and UV–Vis absorption spectra of the Eu-CDs–Fe^3+^ system were recorded for quantification. For portable detection, Eu-CDs@DPPG was immersed in Fe^3+^ solutions of varying concentrations for 10 min. The hydrogel was then placed under a 365 nm UV lamp in a dark box, and a smartphone was fixed in position to capture images. A color recognition app (ColorDesk, V2.21) was used to extract fluorescence color information and convert it into RGB values for further analysis.

### 3.6. Fabrication of Eu-CDs@DPPG Hydrogel

In line with our previous method, Eu-CDs@DPPG was synthesized via free radical polymerization. After dispersing Eu-CDs powder in ultrapure water, acrylamide (AM), and 2-acrylamido-2-methyl-1-propanesulfonic acid (AMPS) were added to the dispersion while stirring. In the pH-neutral solution, 1,6-hexanediol diacrylate (HDDA) and potassium persulfate (KPS) were added. The hydrogel composite material was obtained by stirring polymerization at 45 °C in a nitrogen environment for 4 h.

### 3.7. Pretreatment of Real Samples

To assess the practical applicability of this method, Fe^3+^ was determined by using the standard addition method in spinach and urine. Using urine samples collected from healthy volunteers, centrifugation at 10,000 rpm for five minutes, and filtering the supernatant using a 0.22 μm nylon membrane, the filtrate was diluted 100 times with ultrapure water. For spinach sample pretreatment, fresh spinach leaves were washed, dried, cut into pieces, placed in a microwave digestion tank and heated at 190 °C for 20 min, and then placed on a heating plate and heated at 100 °C for 30 min. The residual solids were fixed to 50 mL with ultrapure water and diluted 20 times for use [39]. After adding different concentrations of Fe^3+^ (0–2.5 μM) to the pretreated samples, the Fe^3+^ concentration was calculated according to the standard curve and the relative standard deviation (RSD) was calculated.

## 4. Conclusions

In summary, we successfully developed a portable, selective, and thermally degradable fluorescent sensor (Eu-CDs@DPPG) for rapid Fe^3+^ detection. The fluorescence of Eu-doped carbon dots (Eu-CDs), serving as the sensing unit, was effectively quenched by Fe^3+^ through the inner filter effect (IFE), enabling the sensitive and selective detection of Fe^3+^ in the urine and spinach sample. Embedding Eu-CDs into a thermally degradable hydrogel matrix, the sensor allowed for visual and quantitative detection of Fe^3+^ using a smartphone-based LAB color recognition system. Furthermore, the hydrogel platform could readily degrade into liquid form at 80 °C, eliminating secondary environmental pollution and facilitating field deployment. This work not only provides a novel sensing strategy for Fe^3+^ but also offers a promising approach to environmentally friendly heavy metal ion detection in future practical scenarios.

## Figures and Tables

**Table 1:** Determination of Fe^3+^ in real samples (n = 3).

Sample	Added (μM)	Found (μM)	Recovery (%)	RSD (%)
spinach	0.5	0.54	108.00	1.95
	1.5	1.53	101.68	1.05
	2.5	2.52	100.70	1.35
urine	0.5	0.51	101.63	1.15
	1.5	1.51	100.46	2.17
	2.5	2.47	98.70	1.50

## Data Availability

Data are contained within the article.

## Supplementary Materials

The following supporting information can be downloaded at: https://www.mdpi.com/article/10.3390/molecules30153280/s1. Figure S1 Optimization of conditions for preparing Eu-CDs: (a) temperature, (b) time. Figure S2 (a) UV-vis, fluorescence excitation spectra and emission spectra of Eu-CDs; (b) Fluorescence excitation spectra of Eu-CDs in the range of 300–400 nm. Figure S3 Stability tests of Eu-CDs (a) NaCl stability, (b) temperature stability, (c) time stability against photobleaching, and (d) pH stability. Figure S4 Anti-interference test of Eu-CDs-Fe3+ system (a) metal ions and acid radicals, (b) pH. Figure S5 Response time test of the Eu-CDs-Fe3+ system. Figure S6 Absorption spectrum of Fe3+ and fluorescence excitation spectrum of Eu-CDs. Figure S7 Images of E-CDs and Eu-CDs@DPPG under 365 nm UV excitation. Table S1. Fluorescence lifetime data statistics (n = 3). Table S2. Parameters used to calculate IFE. Table S3 Comparison of methods. Table S4. Analysis of R G B and L A B values of E-CDs and Eu-CDs@DPPG. References [40,41,42] are cited in Supplementary Materials.
