# Supplementary material for: A Portable and Thermally Degradable Hydrogel Sensor Based on Eu-Doped Carbon Dots for Visual and Ultrasensitive Detection of Ferric Ion
Source: Molecules. 2025 Aug 5;30(15):3280. doi: 10.3390/molecules30153280 (PMC12348846; doi:10.3390/molecules30153280)
Supplement: Supplementary file 1 [file molecules-30-03280-s001.zip › molecules-3798398-supplementary.pdf]

A Portable and Thermally Degradable Hydrogel Sensor Based on Eu-Doped Carbon Dots for Visual and Ultrasensitive Detection of Ferric Ion

Hongyuan Zhang [1,*], Qian Zhang [1], Juan Tang [1], Huanxin Yang [1], Xiaona Ji [1], Jieqiong Wang [2,*], Ce Han [3,*]

[1] School of Science, Changchun Institute of Technology, 395 Kuanping Road, Changchun 130012, China; 22080411109@stu.ccit.edu.cn (Q.Z.); lx_tj@ccit.edu.cn (J.T.); 0207061@ccit.edu.cn (H.Y.); lx_jxn@ccit.edu.cn (X.J.)

[2] School of Materials Science and Engineering, Changchun University, 6543, Weixing Road, Changchun 130022, China;

[3] State Key Laboratory of Electroanalytical Chemistry, Changchun Institute of Applied Chemistry, Chinese Academy of Sciences, Changchun, 130022, China;

* Correspondence: zhanghongyuan@ccit.edu.cn (H.Z.); wangjq94@ccu.edu.cn (J.W.); hance@ciac.ac.cn (C.H.);

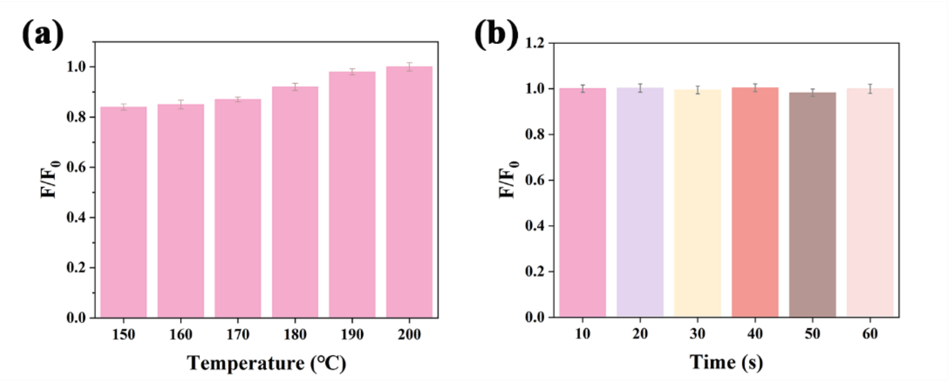

Figure S1. Optimization of conditions for preparing Eu-CDs: (a) temperature, (b) time.

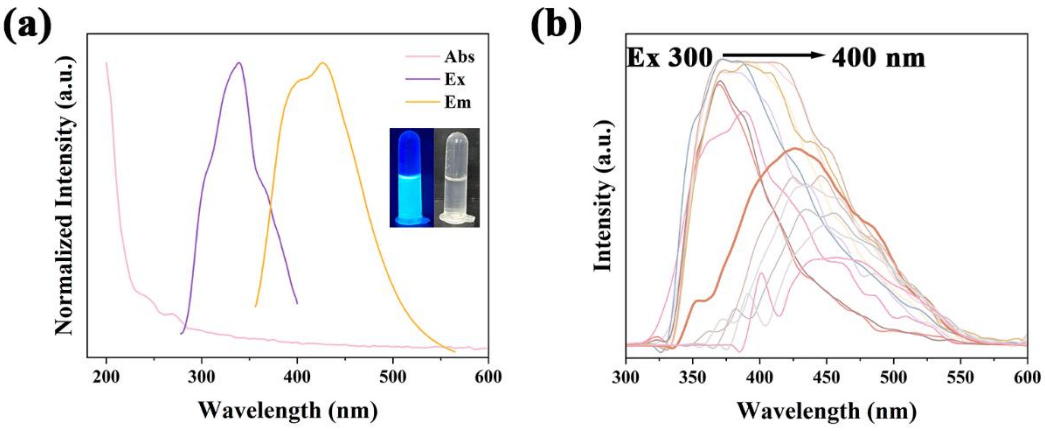

Figure S2. (a) UV-vis, fluorescence excitation spectra and emission spectra of Eu-CDs; (b) Fluorescence excitation spectra of Eu-CDs in the range of 300-400 nm.

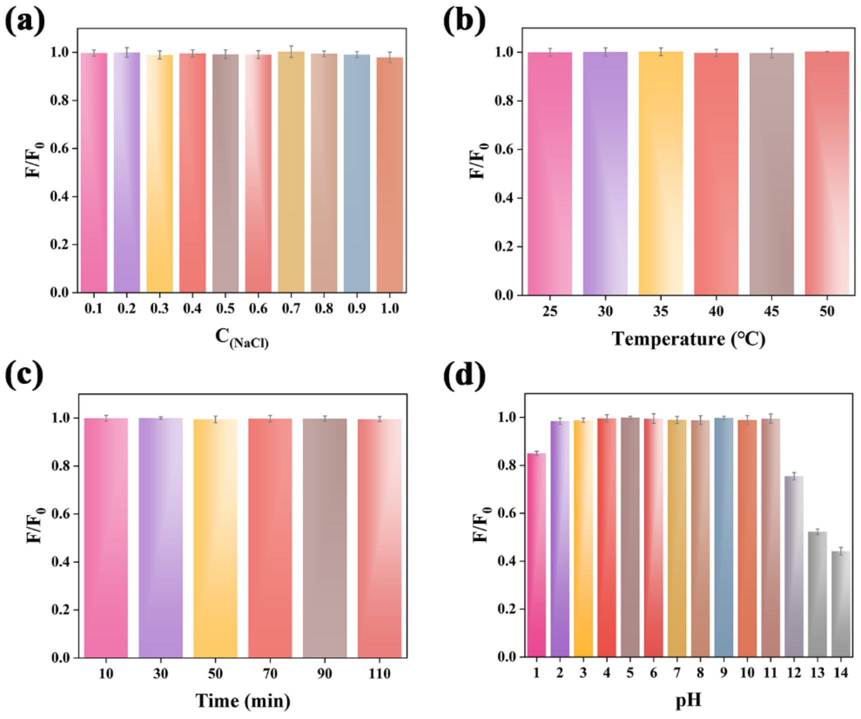

Figure S3. Stability tests of Eu-CDs (a) NaCl stability, (b) temperature stability, (c) time stability against photobleaching, and (d) pH stability.

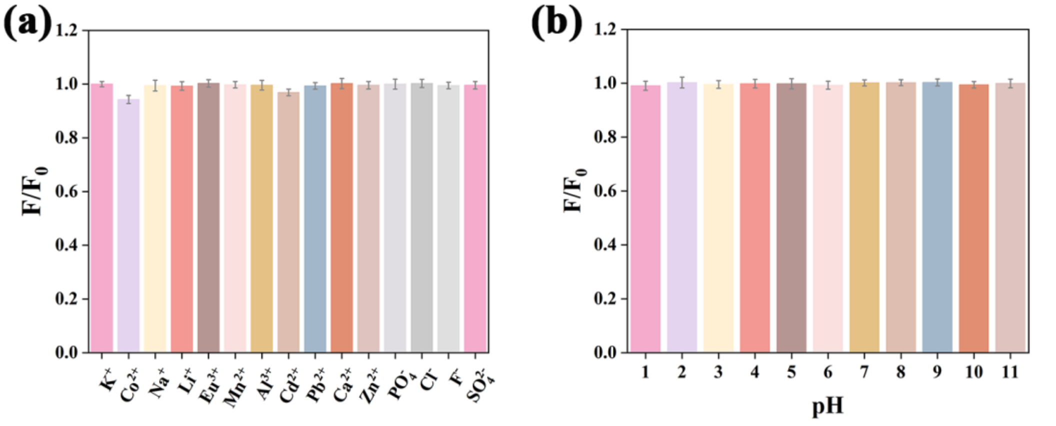

Figure S4 . Anti-interference test of Eu-CDs-Fe^{3+}system (a) metal ions and acid radicals, (b) pH.

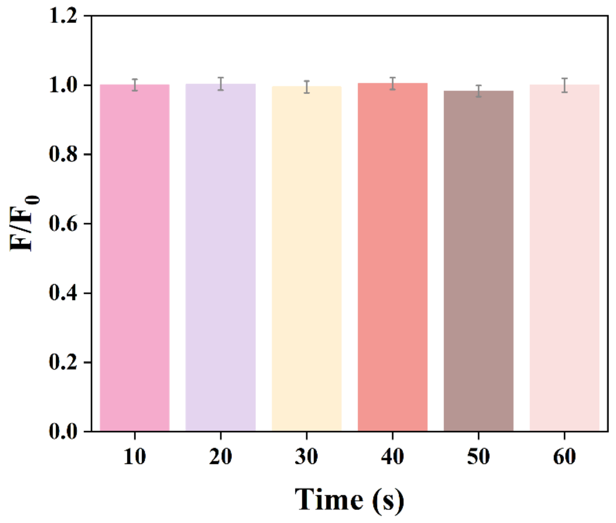

Figure S5. Response time test of Eu-CDs-Fe^{3+} system

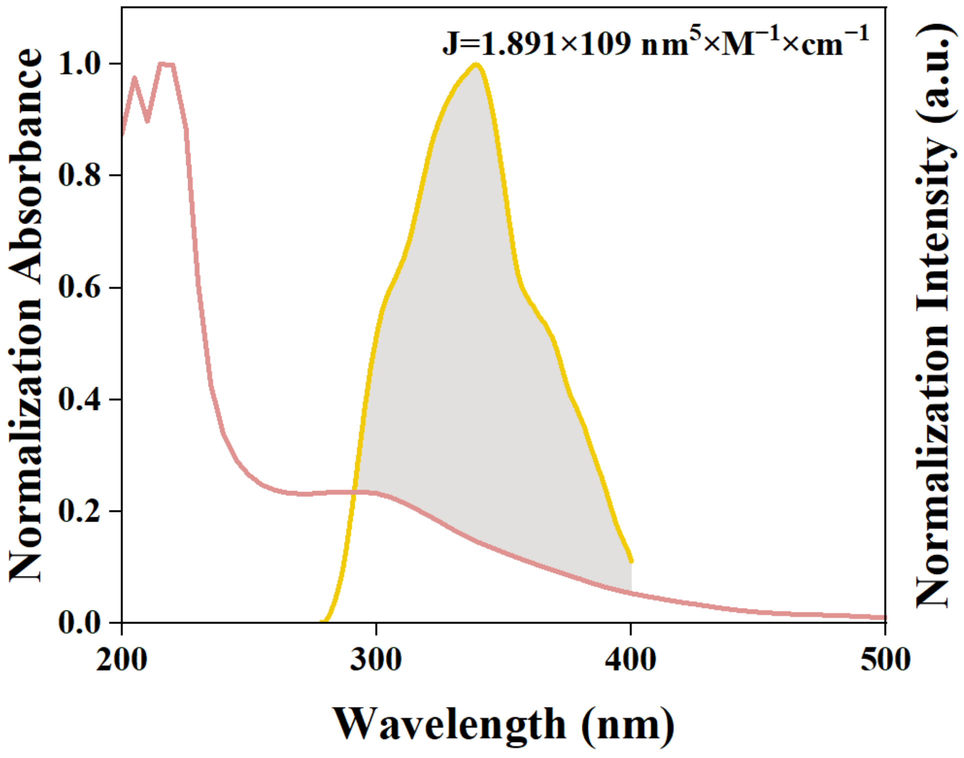

Figure S6. Absorption spectrum of Fe^{3+} and fluorescence excitation spectrum of Eu-CDs Equation of the overlap integral calculation:

$$J = \int \varepsilon(\lambda) \times f_D(\lambda) \times \lambda^4 \, d\lambda \tag{S1}$$

where $\varepsilon(\lambda)$ was the molar absorptivity, $f_D(\lambda)$ was the normalized intensity of the donor emission spectrum, λ was the wavelength, λ^4 was used to quantify the enhancement weight of IFE, and $d\lambda$ was the wavelength interval width.

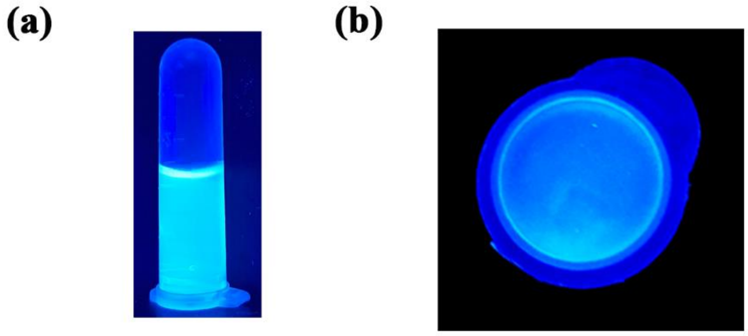

Figure S7. Images of E-CDs and Eu-CDs@DPPG under 365 nm UV excitation

Parker equations S1, S2, and S3:

$$\frac{F_{cor}}{F_{obsd}} = \frac{2.3dA_{ex}}{1-10^{-dA_{ex}}} 10^{gA_{em}} \frac{2.3sA_{em}}{1-10^{-sA_{em}}} \quad \text{(S2)}$$

$$E_{obsd} = 1 - \frac{F_{obsd}}{F_{obsd,0}} \quad \text{(S3)}$$

$$E_{cor} = 1 - \frac{F_{cor}}{F_{cor,0}} \quad \text{(S4)}$$

F_{obsd} and F_{cor} represented the measured and corrected fluorescence intensities, respectively. The E_{obsd} and E_{cor} were the measured and corrected fluorescence intensity changes. A_{ex} and A_{em} denoted the absorbance at the optimal excitation wavelength (340 nm) and optimal emission wavelength (426 nm), respectively. “d” was the width of the cuvette and ‘s’ was the distance between the edge of the cuvette and the edge of the excitation beam.

Table S1. Fluorescence lifetime data statistics (n=3)

Sample	average values	standard deviations (SD)	relative standard deviations (RSD)	t-values	p-values
Eu-CDs	3.71×10^{-9}	9.05×10^{-11}	2.50%	0.400	0.716
Eu-CDs-Fe^{3+}	3.59×10^{-9}	1.69×10^{-10}	4.73%		

Table S2. Parameters used to calculate IFE

RES (mg/mL)	A_{ex} (340 nm)	A_{em} (426 nm)	F_{cor}/F_{obsd}	E_{obsd}	E_{cor}
0.00	1.389	0.435	3.466	0	0
0.02	1.392	0.438	3.475	0.011	0.013
0.04	1.407	0.503	3.591	0.172	0.200
0.06	1.484	0.510	3.779	0.192	0.259
0.08	1.548	0.516	3.943	0.209	0.305
0.10	1.654	0.531	4.196	0.242	0.374

Table S3. Comparison of methods.

Materials|methods	Sample	Linear range	LOD	Refs
ultrathin boron nanosheets|FL	human serum	0.2-150 μM	20 nM	[1]
hybrid iron oxide/carbon dots (HICs) |FL	Milk and herbal drinks	0.06-100 μM	13.61 nM	[2]
UiO-66-NH_2@BPEA-NF|FL	environmental water, human serum and cell lysates	0.5-5 μM	175 nM	[3]
BP-N-CDs|FL	human serum and urine	0-2.5 μM	1.25 nM	This work

Table S4. Analysis of R G B and L A B values of E-CDs and Eu-CDs@DPPG

Sample	L Value	A Value	B Value	R Value	G Value	B Value	Average value	RSD （%）
Eu-CDs	81	-38	-30	0	222	255	R=0.200<br>G=216.200<br>B=254.600<br>L=79.200<br>A=-35.600<br>B=-32.000	R=0<br>G=2.053<br>B=0.215<br>L=1.646<br>A=-5.103<br>B=-4.941
	79	-35	-32	0	216	255		
	78	-36	-33	0	213	254		
	80	-36	-31	0	219	255		
	78	-33	-34	0	211	254		
Eu-CDs@DPPG	75	-39	-38	0	202	254	R=0.400<br>G=199.800<br>B=254.000<br>L=74.400<br>A=-30.400<br>B=-39.200	R=136.9<br>G=2.610<br>B=0<br>L=2.038<br>A=-4.857<br>B=-6.603
	72	-35	-43	1	192	254		
	75	-39	-39	0	201	254		
	76	-36	-36	0	206	254		
	74	-38	-40	1	198	254		

References

1. Guo, T.; Xu, J.; Guo, Y. T.; Ning, B.; Li, J. T.; Gong, L. Z.; Lin, X. Y.; Zhuang, S. H.; Wei, Z. W., Ultrathin boron nanosheets: a novel fluorescent sensor for sensitive and selective detection of Fe3+and ascorbic acid. *SPECTROCHIMICA ACTA PART A-MOLECULAR AND BIOMOLECULAR SPECTROSCOPY* **2025,** 343.
2. Nuntahirun, P.; Li, C. H.; Sirisit, N.; Shashikumar, U.; Tsai, P. C.; Manjappa, K. B.; Huang, G. G.; Paoprasert, P.; Ponnusamy, V. K., Novel blue-pea flowers derived-carbon dots/iron oxide nanohybrid as sustainable "turn-off" fluorescent nanosensor for selective Fe3+detection in food samples. *SPECTROCHIMICA ACTA PART A-MOLECULAR AND BIOMOLECULAR SPECTROSCOPY* **2025,** 339.
3. Zhang, Q. K.; Dou, S. H.; Leng, H.; Shu, Y., A small molecule modified UiO series MOFs for simultaneous detection of Fe³⁺ and Zn²⁺. *TALANTA* **2025,** 286.
